# Progressive multifocal leukoencephalopathy in a young adult with *DOCK8* deficiency: a case of JC virus reactivation in primary immunodeficiency

**DOI:** 10.1007/s13365-025-01279-2

**Published:** 2025-11-12

**Authors:** Laura Naydovich, Joseph R. Berger, Pavle S. Milutinovic

**Affiliations:** 1 Department of Neurology, Division of Multiple Sclerosis, University of Pennsylvania, Philadelphia, PA USA; 2 Department of Medicine, Division of Pulmonary, Allergy, and Critical Care Medicine, University of Pennsylvania, Philadelphia, PA USA

**Keywords:** Progressive multifocal leukoencephalopathy, JC virus reactivation, DOCK8 deficiency, Primary immunodeficiency, Hyper-IgE syndrome

## Abstract

Progressive multifocal leukoencephalopathy (PML) is a rare, often fatal demyelinating disease of the central nervous system (CNS) caused by JC virus (JCV) reactivation in the setting of impaired cellular immunity. While commonly associated with human immunodeficiency virus/acquired immunodeficiency syndrome (HIV/AIDS) and immunosuppressive therapies, PML can also arise in primary immunodeficiency disorders. We report a case of PML in a young adult with autosomal recessive hyper-IgE syndrome (AR-HIES) due to *DOCK8* deficiency, highlighting the importance of considering genetic immunodeficiencies in cases of JCV-PML without known iatrogenic or acquired causes.

## Background

*DOCK8* deficiency is a rare autosomal recessive combined immunodeficiency caused by pathogenic variants in *DOCK8* (Engelhardt et al. 2009; Zhang et al. 2009), a gene encoding a guanine nucleotide exchange factor expressed in B and T lymphocytes and hematopoietic tissues including the placenta, kidney, lung and pancreas (Ruusala and Aspenström 2004). *DOCK8* is essential for actin cytoskeleton regulation and immune cell trafficking and survival (Zhang et al. 2014). Its deficiency is the most common underlying pathology of autosomal recessive hyper-IgE syndrome (AR-HIES) (Engelhardt et al. 2009; Renner et al. 2004). Affected individuals typically present in childhood or young adulthood with recurrent viral, bacterial, and fungal infections, elevated serum IgE, atopy, and autoimmunity (Zhang et al. 2009; Renner et al. 2004; Aydin et al. 2015). Neurotropic herpetic infections such as HSV and VZV are common, while JCV infection is rare but has been reported (Engelhardt et al. 2009; Day-Williams et al. 2015; Soldatos et al. 2022). CNS complications in *DOCK8* deficiency may include both infectious (e.g., meningitis, encephalitis) and non-infectious manifestations (e.g., vasculitis, aneurysms, stroke, and brain and optic atrophy), with the latter reflecting a broader neuroinflammatory and vascular vulnerability independent of infection (Engelhardt et al. 2009; Zhang et al. 2009; Aydin et al. 2015). There is an increased risk of malignancy, particularly hematological and cutaneous neoplasms (Engelhardt et al. 2009; Aydin et al. 2015). Diagnosis is based on clinical features, laboratory findings (including elevated serum IgE), and genetic confirmation. Management includes antimicrobial prophylaxis, surveillance for infections and malignancy, and in eligible cases, hematopoietic stem cell transplantation (HSCT), which is potentially curative (Biggs et al. 2017; Cuellar-Rodriguez et al. 2015).

## Case report

A 21-year-old man from Dubai with a history of glucose-6-phosphate-dehydrogenase deficiency, thalassemia trait, recurrent sinopulmonary infections, bronchiectasis, eczema, warts, severe chickenpox, and prior herpetic keratitis requiring corneal transplant. He was put on antimicrobial prophylaxis (oral cephalosporin) after immunological workup revealed CD4 + lymphopenia, low IgM, elevated IgE, and poor vaccine response. He was not offered immunoglobulin replacement therapy.

**Fig. 1:**
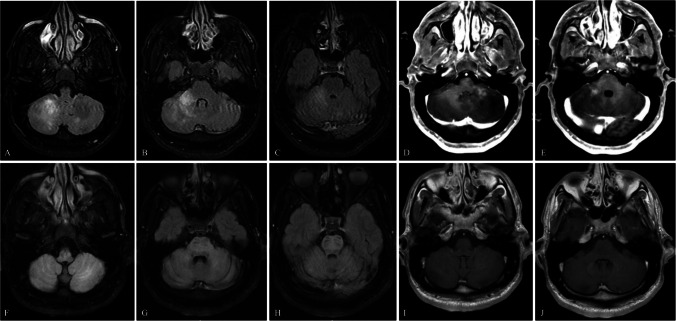
**A**-**E**: MRI brain with and without contrast at time of PML symptom onset (October 2024). Images **A**-**C** show axial T2/FLAIR signal abnormalities. Images **D**, **E** show axial T1 post-contrast enhancement. **G**-**J**: MRI brain with and without contrast at time of PML symptom stability (April 2025). Images **F**–**H** show axial T2/Flair signal abnormalities. Images **D**, **E** show axial T1 post-contrast enhancement resolution

In October 2024, he presented with 2-month history of progressive gait dysfunction, dysarthria, dysphagia, and left face and arm numbness over two months. MRI revealed gadolinium-enhancing T2/Fluid-Attenuated Inversion Recovery (FLAIR) hyperintensities in the cerebellum and brainstem (see Fig. 1). JCV polymerase chain reaction (PCR) of cerebrospinal fluid (CSF) revealed 5,320 copies/mL. A diagnosis of definite PML (Berger et al. 2013) was established based on clinical, radiographic and CSF JCV PCR results.

In the absence of any therapy, he experienced partial, spontaneous improvement in dysarthria, dysphagia, and numbness in the absence of treatment. However, his incoordination and gait dysfunction persisted six months from symptom onset, prompting presentation to the University of Pennsylvania for further workup and management of JCV-PML and immunodeficiency. Neurological examination showed intact mental status, fluent language, ataxic dysarthria, bilateral optic disc pallor, retinal vascular sheathing, and ocular dysmetria. Remainder of the cranial nerves were normal. Frontal release signs were absent. Muscle tone and bulk and confrontational strength were normal, although he exhibited satelliting about the left arm. There was mild-moderate ataxia in all extremities, greater on the right. Reflexes were 2 + in the upper extremities, 3 + in the lower extremities with right ankle clonus and a positive right Babinski. Sensation to pinprick, vibration, and light touch were preserved. He required assistance walking due to gait ataxia and found it difficult to stand independently on a narrow base.

Repeat cranial MRI on April 17, 2025 demonstrated a modest decrease in the T2/FLAIR signal in the right cerebellar hemisphere and resolution of previously noted abnormal enhancement (see Fig. 1). Laboratory workup showed CD4 lymphopenia, normal IgG and IgA, low IgM at < 10 mg/dL, and high IgE at 2916 mg/dL. Whole genome sequencing identified compound heterozygous pathogenic variants in *DOCK8*, including a deletion of exons 15–25 and a frameshift mutation (c.4957_4958del; p.Thr1653Glnfs*21), consistent with AR-HIES. A third variant in the same gene (c.5408A > G; p.Lys1803Arg) was of uncertain significance. Flow cytometry confirmed nearly absent *DOCK8* protein expression in monocytes and B cells, partially decreased in NK cells, with marginally decreased protein expression in CD3 + T cells.

Given his clinical improvement and radiological stability, experimental treatment with pembrolizumab was deferred. It would not be unexpected for patients with *DOCK8* deficiency to have succumbed to severe infection or malignancy by his age. His favorable course may reflect somatic reversion in NK and/or T cells, mitigating the impact of *DOCK8* deficiency (Pillay et al. 2021; Jing et al. 2014) and accounting for his survival to this point on suboptimal prophylaxis. He was initiated on prophylactic sulfamethoxazole-trimethoprim, valacyclovir, and IVIG. He did not require antifungal prophylaxis. He was referred for HSCT consideration through the National Institutes of Health and National Institute of Allergy and Infectious Disease where hematopoietic stem cell transplantation (HSCT) is under consideration if his PML remains stable or improved.

## Discussion

We describe the occurrence of virus PML in a young adult with *DOCK8* deficiency—a form of AR-HIES marked by combined immunodeficiency and viral susceptibility. Two similar cases have been reported: a 30-year-old man with homozygous deletions of *DOCK8* on chromosome 9p who developed PML with progressive neurological decline and seizures (Day-Williams et al. 2015) and a neurologically asymptomatic 21-year-old man with cytotoxic T-cell lymphoma and *DOCK8* deficiency (variant not detailed) in whom PML was detected and was managed with HSCT (Soldatos et al. 2022). The apparent stability of our patient’s PML may be the consequence of a somatic reversion in NK and/or T cells. More broadly, this case underscores PML as a rare but important manifestation of primary immunodeficiency. In young patients with JCV-related CNS disease and no acquired immunosuppression, inborn errors of immunity should be considered even in adults. Early recognition, supportive care, and emerging immunotherapies are critical for optimizing outcomes.

## Data Availability

No datasets were generated or analysed during the current study.

## References

[CR1] Aydin SE, Kilic SS, Aytekin C, Kumar A, Porras O, Kainulainen L et al (2015) DOCK8 deficiency: clinical and immunological phenotype and treatment options - a review of 136 patients. J Clin Immunol 35(2):189–198. 10.1007/s10875-014-0126-0 25627830 10.1007/s10875-014-0126-0

[CR2] Berger JR, Aksamit AAJ, Clifford DB, Davis L, Koralnik IJ, Sejvar JJ, Bartt R, Major EO, Nath A (2013) PML diagnostic criteria: consensus statement from the AAN neuroinfectious disease section. Neurology 80(15):1430–1438 23568998 10.1212/WNL.0b013e31828c2fa1 PMC3662270

[CR3] Biggs CM, Keles S, Chatila TA (2017) DOCK8 deficiency: insights into pathophysiology, clinical features and management. Clin Immunol 181:75–82. 10.1016/j.clim.2017.06.003 28625885 10.1016/j.clim.2017.06.003 PMC5555255

[CR4] Cuellar-Rodriguez J, Freeman AF, Grossman J, Su H, Parta M, Murdock H et al (2015) Matched related and unrelated donor hematopoietic stem cell transplantation for DOCK8 deficiency. Biol Blood Marrow Transplant 21:1037–1045. 10.1016/j.bbmt.2015.01.022 25636378 10.1016/j.bbmt.2015.01.022 PMC4426076

[CR5] Day-Williams AG, Sun C, Jelcic I, McLaughlin H, Harris T, Martin R et al (2015) Whole genome sequencing reveals a chromosome 9p deletion causing DOCK8 deficiency in an adult diagnosed with hyper IgE syndrome who developed progressive multifocal leukoencephalopathy. J Clin Immunol 35(1):92–96. 10.1007/s10875-014-0114-4 25388448 10.1007/s10875-014-0114-4 PMC4306731

[CR6] Engelhardt KR, McGhee S, Winkler S, Sassi A, Woellner C, Lopez-Herrera G et al (2009) Large deletions and point mutations involving the dedicator of cytokinesis 8 (DOCK8) in the autosomal-recessive form of hyper-IgE syndrome. J Allergy Clin Immunol 124(6):1289–1302. 10.1016/j.jaci.2009.10.038 20004785 10.1016/j.jaci.2009.10.038 PMC2818862

[CR7] Jing H, Zhang Q, Zhang Y, Hill BJ, Dove CG, Gelfand EW et al (2014) Somatic reversion in dedicator of cytokinesis 8 immunodeficiency modulates disease phenotype. J Allergy Clin Immunol 133(6):1667–1675. 10.1016/j.jaci.2014.03.025 24797421 10.1016/j.jaci.2014.03.025 PMC4132167

[CR8] Pillay BA, Fusaro M, Gray PE, Statham AL, Burnett L, Bezrodnik L et al (2021) Somatic reversion of pathogenic DOCK8 variants alters lymphocyte differentiation and function to effectively cure DOCK8 deficiency. J Clin Invest 131(3):e142434. 10.1172/JCI142434 33290277 10.1172/JCI142434 PMC7843233

[CR9] Renner ED, Puck JM, Holland SM, Schmitt M, Weiss M, Frosch M et al (2004) Autosomal recessive hyperimmunoglobulin E syndrome: a distinct disease entity. J Pediatr 144(1):93–99. 10.1016/S0022-3476(03)00449-9 14722525 10.1016/S0022-3476(03)00449-9

[CR10] Ruusala A, Aspenström P (2004) Isolation and characterisation of DOCK8, a member of the DOCK180-related regulators of cell morphology. FEBS Lett 572(1–3):159–166. 10.1016/j.febslet.2004.06.095 15304341 10.1016/j.febslet.2004.06.095

[CR11] Soldatos A, Cortese I, Fletcher A, Gonzalez C, Freeman A (2022) A tale of 2 leukodystrophies in DOCK8 deficiency, a rare primary immunodeficiency (P17–1.002). Neurology 98(18 Suppl):57635190464

[CR12] Zhang Q, Davis JC, Lamborn IT, Freeman AF, Jing H, Favreau AJ et al (2009) Combined immunodeficiency associated with DOCK8 mutations. N Engl J Med 361:2046–2055. 10.1056/NEJMoa0905506 19776401 10.1056/NEJMoa0905506 PMC2965730

[CR13] Zhang Q, Dove CG, Hor JL, Murdock HM, Strauss-Albee DM, Garcia JA et al (2014) DOCK8 regulates lymphocyte shape integrity for skin antiviral immunity. J Exp Med 211(13):2549–2566. 10.1084/jem.20141307 25422492 10.1084/jem.20141307 PMC4267229

